# Comparative Study of Phenolic Content and Antioxidant and Hepatoprotective Activities of Unifloral Quillay Tree (*Quillaja saponaria* Molina) and Multifloral Honeys from Chile

**DOI:** 10.3390/plants13223187

**Published:** 2024-11-13

**Authors:** Paula Núñez-Pizarro, Gloria Montenegro, Gabriel Núñez, Marcelo E. Andia, Christian Espinosa-Bustos, Adriano Costa de Camargo, Juan Esteban Oyarzún, Raquel Bridi

**Affiliations:** 1 Departamento de Ciencias Vegetales, Facultad de Agronomía y Sistemas Naturales, Pontificia Universidad Católica de Chile, Santiago 7820436, Chile; pjnunez@uc.cl (P.N.-P.); gmonten@uc.cl (G.M.); ginunez@uc.cl (G.N.); 2 Biomedical Imaging Center, School of Medicine, Pontificia Universidad Católica de Chile, Santiago 3580000, Chile; meandia@uc.cl; 3 ANID-Millennium Institute for Intelligent Healthcare Engineering—iHEALTH, Pontificia Universidad Católica de Chile, Santiago 7820436, Chile; 4 Departamento de Farmacia, Facultad de Química y de Farmacia, Pontificia Universidad Católica de Chile, Santiago 7820436, Chile; ccespino@uc.cl; 5 Nutrition and Food Technology Institute, University of Chile, Santiago 7830490, Chile; adrianodecamargo@inta.uchile.cl; 6 Departamento de Química Farmacológica y Toxicológica, Facultad de Ciencias Químicas y Farmacéuticas, Universidad de Chile, Santiago 8380000, Chile

**Keywords:** soapbark tree, antioxidants, cytotoxicity, hepatoprotection, polyphenols

## Abstract

Honey is a natural sweet element that bees make with flower nectar, revered for its distinct flavor, nutritional value, and potential health benefits. Chilean beekeeping has a diverse range of honey varieties, many of which are unique. The quillay (*Quillaja saponaria* Molina, soapbark tree) is a Chilean endemic tree whose honey has not been studied in depth. We characterized various Chilean honeys with different botanical origins, with a particular focus on quillay tree honey, analyzing its total phenolic and flavonoid content and its antioxidant activities. Cytotoxicity and hepatoprotective activity were also evaluated using HuH-7 cells. The Spearman correlation between the percentage of quillay pollen in the honey samples and the total phenolic content (R = 0.72; *p* < 0.05), plus the oxygen radical absorbance capacity, suggests that compounds from quillay contribute to the overall antioxidant capacity of honey. Unifloral quillay honey extracts also protect hepatic cells from oxidative damage induced by peroxyl radicals generated by AAPH. This analysis sheds light on the potential of quillay tree honey, underscoring its significance as a natural source of bioactive phenolic compounds with possible hepatoprotective effects.

## 1. Introduction

Honey is a natural complex product comprising several compounds: sugars like glucose and fructose (60–85%), proteins (between 0.2% and 1.6%) and amino acids (alanine, phenylalanine, glutamic acid, leucine, tyrosine, proline), enzymes (invertase, catalase, acid phosphatase, diastase, glucose oxidase), and other components such as phenolic compounds (phenolic acids and flavonoids), minerals (sodium, magnesium, calcium, iron), and vitamins (mainly vitamin B complex and vitamin C) [1,2,3,4]. Honey has been consumed since prehistoric times, and the most ancient populations used honey for its nutritional and therapeutical properties [5].

Foraging bees, particularly *Apis mellifera*, take nectar from flowers they visit and bring it back home in their crop (honey stomach), where enzymatic processing initiates the metabolization of nectar components [6]. The nectar is later deposited into honeycomb cells in their hive, and workers promote water removal [7]. When the bees realize that honey is ready for storage, they seal the cell by applying a layer of wax [8].

Honey has a long history of medicinal use, and research has demonstrated that it has antimicrobial, anti-inflammatory, immune-boosting, anticancer, and antioxidant effects [1,9,10,11,12]. The antioxidant capacity of natural products is linked to various biological activities, including hepatoprotective potential. Excessive reactive oxygen species (ROS) can damage cellular components, such as lipids, proteins, and DNA, triggering inflammation, apoptosis, and tissue injury [13]. The liver, an essential organ for metabolic and detoxification functions, is vulnerable to damage from ROS accumulation, leading to oxidative stress and contributing to the progression of chronic liver diseases, such as cirrhosis, non-alcoholic fatty liver disease (NAFLD), alcoholic liver disease (ALD), and hepatocellular carcinoma [14]. Chronic liver conditions consistently exhibit increased oxidative stress, regardless of the underlying cause [15,16]. The antioxidant properties of honey are particularly relevant in liver diseases, as reducing ROS helps mitigate oxidative tissue damage [16]. Understanding the equilibrium between ROS and antioxidants is essential to understand the mechanisms underlying liver diseases and study the potential of honey as a hepatoprotective agent. The capacity of honey to diminish oxidative stress makes it a promising candidate for further investigation in preventing and treating liver disorders [17].

Various factors affect honey’s quality and composition, including its floral origin, soil and weather conditions, and geographical location [18,19]. Among these, the presence of secondary metabolites such as phenolic compounds plays an important role, as they are transferred from plants through bee activity [20,21,22]. Identifying phenolic acids and flavonoids associated with the floral source of honey can add value by certifying its botanical origin and linking it to specific chemical and biological properties.

Chilean honey is characterized by its diverse biological properties arising from its unique chemical composition [23]. Phenolic compounds such as gallic, caffeic, and *p*-coumaric acids, along with flavonoids like pinocembrin, chrysin, quercetin, galangin, kaempferol, and apigenin, act as distinctive markers of honey, contributing to its antioxidant, antimicrobial, and anti-inflammatory properties [24,25,26]. Quillay (*Quillaja saponaria* Molina, also known as soapbark tree) is an endemic tree of Chile, found in the sclerophyllous forests of central Chile [26], between 30°30′ and 38° S, spanning from the Coquimbo Region (IV) to the Araucanía Region (IX) [27,28,29,30]. This species primarily develops in the central zone of Chile, thriving between the eastern slope of the Coastal Mountain Range and the western slope of the Andes, where a Mediterranean climate predominates (30° to 36° S) [28,31]. Temperatures in this region range from −3.2 °C to 9.4 °C in winter and 16.5 °C to 31.3 °C in summer, with an average annual temperature of approximately 14 °C [27]. The quillay tree is one of the most significant melliferous species in the country [29,32]. Previous studies have shown that its flowers have large nectaries correlating with high nectar production, explaining its significant contribution to Chilean unifloral honey [28,29]. It has been shown that both quillay tree honey and its extracts have antioxidant capacity related to the presence of phenolic compounds [33]. Analytical methods such as HPLC-DAD showed the presence of chlorogenic acid, caffeic acid, syringic acid, *p*-coumaric acid, gallic acid, myricetin, rutin, quercetin, and naringenin in both quillay nectar and honey [32].

The present study explored melissopalynological analysis, total phenolic and flavonoid content, antioxidant capacity, and hepatoprotective activity in HuH-7 cells of Chilean honey samples. This is the first time that Chilean unifloral quillay honey extracts have been studied in hepatic cell cultures using Human Hepatoma cells, HuH-7. Phenolic acid and flavonoid profiles of the quillay floral nectaries and honey samples were described as well.

## 2. Results

### 2.1. Melissopalynological Analysis

Honey samples were obtained from associated beekeepers. The botanical origin was determined following the Chilean Standard NCH 2981.OF2005 [31], which determines the botanical origin of the honey through a melissopalynological test and classifies honeys as unifloral, bifloral, or multifloral [31]. According to this standard, honey is classified as unifloral if over 45% of its pollen grains come from a single plant species. If pollen from two species makes up more than 50% of the total grains, with a difference of at least 5% between them, the honey is considered bifloral. Lastly, when no single species contributes more than 45% of the total pollen grains, the honey is classified as multifloral. The standard also subclassifies samples into endemic/native, non-native, and mixed, depending on the species contributing to honey production [31]. Eleven samples have quillay (*Quillaja saponaria* M.) as a primary or secondary species in their botanical configuration. The percentage of the pollen grains ranged between 17% and 70%. To ensure a comprehensive comparison and validate analytical results, the samples represented multifloral, unifloral, and bifloral honeys with varied botanical compositions. One sample, a non-native unifloral honey with *Lotus pedunculatus* (greater bird’s-foot-trefoil) as the primary species, served as a control and contained no quillay (Sample 1, Table 1). The geographical origin, the three predominant plant species in the samples, and the classification are detailed in Table 1.

### 2.2. HPLC-DAD Analysis, Total Phenolic Content, and Total Flavonoid Content

Table 2 shows a comparable phenolic acid profile between nectaries and honey samples. Table 3 contains the flavonoid profile. For the nectaries, chlorogenic acid, syringic acid, sinapic acid, ferulic acid, and phytohormone abscisic acid were identified, and the flavonoids rutin, quercetin, pinocembrin, chrysin, and genistein were also identified. Within the honey, chlorogenic acid, syringic acid, *p*-coumaric acid, and abscisic acid were found in all samples, and sinapic acid was detected in 10 samples (Table 2). The flavonoids galangin, pinocembrin, and chrysin were detected in all honey samples (Table 3). Sample 11, with 65.4% of quillay, has the highest value for chlorogenic acid (8.58 mg/kg honey), *p*-coumaric acid (0.63 mg/kg honey), ferulic acid (11.36 mg/kg honey), and abscisic acid (56.03 mg/kg honey). Pinocembrin and galangin were found in all honey samples, and sample 12, with the highest quillay pollen percentage (69.8%), showed the highest values for these flavonoids, with 2.24 and 5.49 mg/kg honey, respectively. Abscisic acid (ABA) is a phytohormone primarily involved in plant stress responses and is commonly found in nectar and, consequently, in honey [34,35,36]. This study detected ABA in the nectary extract (33.85 mg/kg) and all honey samples, with an average concentration of 17.4 mg/kg honey.

Chrysin was identified in all honey samples, with a range of 3.54 to 9.91 mg/kg (Table 3). Its highest concentration (9.91 mg/kg honey) was found in the unifloral quillay honey sample 12, which has the most significant quillay pollen level (69.8%). A correlation was observed between the samples’ quillay percentages and their chrysin concentrations (R_Spearman_ = 0.59; Figure S1). It was also detected in the nectaries at a concentration of 1.04 mg/kg. This is the first report on the presence of chrysin in quillay tree honey. Along with this flavonoid, syringic acid was identified in the nectaries (52.67 mg/kg) and in all honey samples (average of 1.11 mg/kg honey), with a Spearman correlation of 0.76 between the percentage of quillay present in the samples and the syringic acid concentrations (Figure S2).

Table 4 shows the total phenolic content, total flavonoid content, and antioxidant activity (FRAP and ORAC-FL) values for the analyzed honey samples. The TPC of honey samples ranged between 52.7 and 85.2 mg GAE/kg honey. Sample twelve presents the highest value for TPC (85.2 mg GAE/kg honey) along with the highest quillay percentage (69.8%), followed by sample 11 (74.8 mg GAE/kg honey). The TFC ranged between 7.4 and 20.7 mg QE/kg honey. Sample 3 has the highest TFC (20.7 mg QE/kg honey), followed by sample 12 (16.8 mg QE/kg honey). TPC and TFC values are similar to those shown in previous publications on Chilean honey [23,24,33,37].

The antioxidant capacity, evaluated by ORAC-FL and FRAP, was between 84.5 and 400 μmol TE/100 g honey and 3.6 and 8.1 μmol TE/100 g honey, respectively. The ORAC values were similar to those previously reported for honeys from the O’Higgins, Maule, Los Lagos, and Metropolitan Regions in Chile [24,33]. The highest value for the FRAP assay was for sample 11 (65.5% quillay), with 8.1 μmol TE/100 g honey, followed by 6.8 μmol TE/100 g honey, corresponding to sample 4.

**Figure 1:**
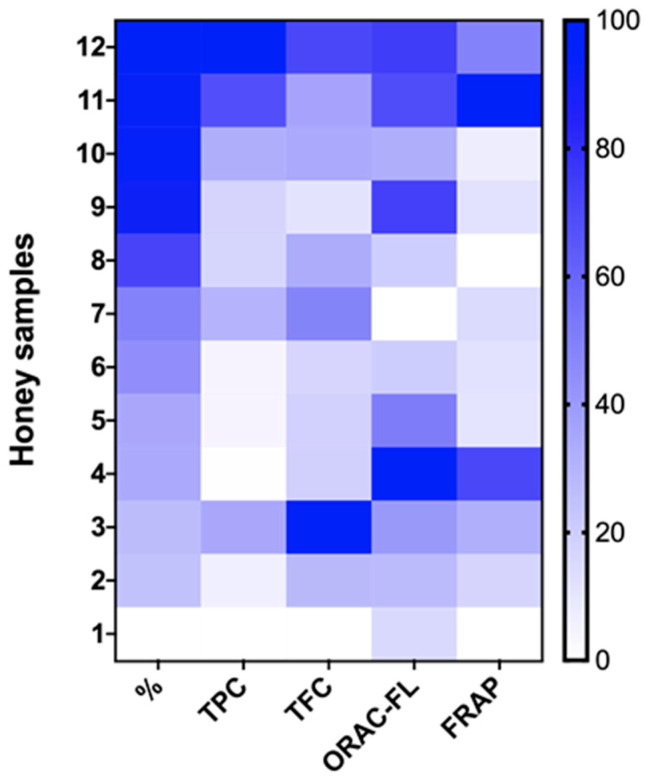
Heat map analysis for parameters studied in all honey samples (%: percentage of quillay pollen; TPC: total phenolic content; TFC: total flavonoid content; ORAC-FL: oxygen radical absorbance capacity; FRAP: ferric reducing antioxidant power).

A heat map was carried out with the percentage of quillay pollen plus the TPC, TFC, ORAC-FL, and FRAP values (Figure 1). The analysis revealed a trend between the presence of quillay in honey samples and the levels of total phenolic content (TPC), total flavonoid content (TFC), and oxygen radical absorbance capacity (ORAC-FL). Samples with a higher percentage of quillay pollen (8–12) tend to have higher phenolic and flavonoid contents and greater antioxidant capacity. Along with this, the Spearman correlation analysis showed a strong positive correlation between the percentage of quillay pollen in every sample and the TPC (R = 0.72; *p* < 0.05), showing a possible influence of *Quillaja saponaria* nectar on the phenolic composition of honey (Figure S3). A strong correlation was visible between TPC and TFC values as well (R = 0.82; *p* < 0.05), suggesting that quillay may contribute to the overall antioxidant capacity of honey, highlighting its potential as a natural source of antioxidants with potential health benefits. On the contrary, samples 1 to 6 have lower TPC, TFC, ORAC- FL, and FRAP values, as indicated by the lighter colors in these map areas.

### 2.3. Cytotoxicity and Hepatoprotective Activity

**Figure 2:**
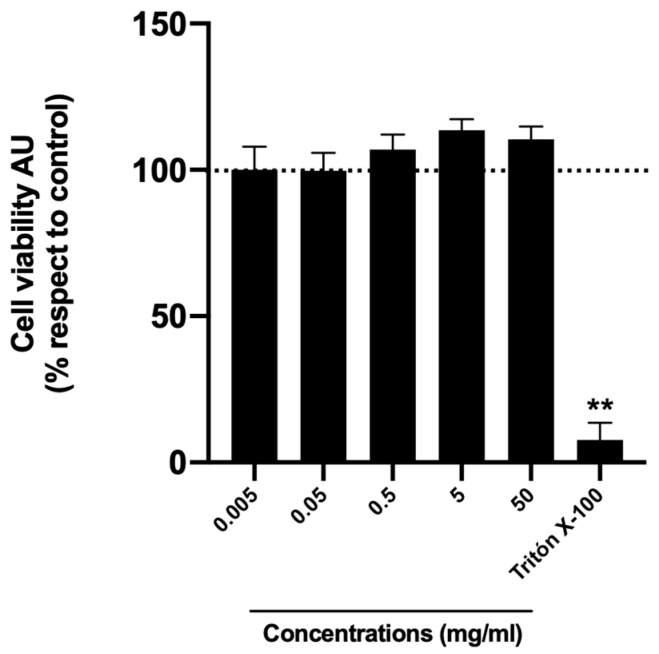
Honey samples’ cytotoxicity. Cell viability was evaluated by Alamar blue of HuH-7 cells treated for 24 h with honey extract of the test sample (sample 9) at different dilutions (0.005, 0.05, 0.5, 5, and 50 mg/mL). To control cell death, cells were treated with Triton X-100 at 1% for 10 min. Data are expressed as the percentage of viability with respect to the control cells. AU, arbitrary units. Data are shown as mean ± SD (n = 3). A one-way ANOVA statistical test was performed, followed by the Tukey test. Statistically significant differences compared to the control group (cells without treatment) (** *p* < 0.01).

To begin, honey cytotoxicity was determined using sample 9. HuH-7 cells were incubated for 24 h using five different extract dilutions (0.005, 0.05, 0.5, 5, and 50 mg/mL). Viability was measured as a percentage relative to the untreated cells (control cells), which were considered to present 100% cell viability with the Alamar blue viability assay. All concentrations were noncytotoxic (Figure 2).

**Figure 3:**
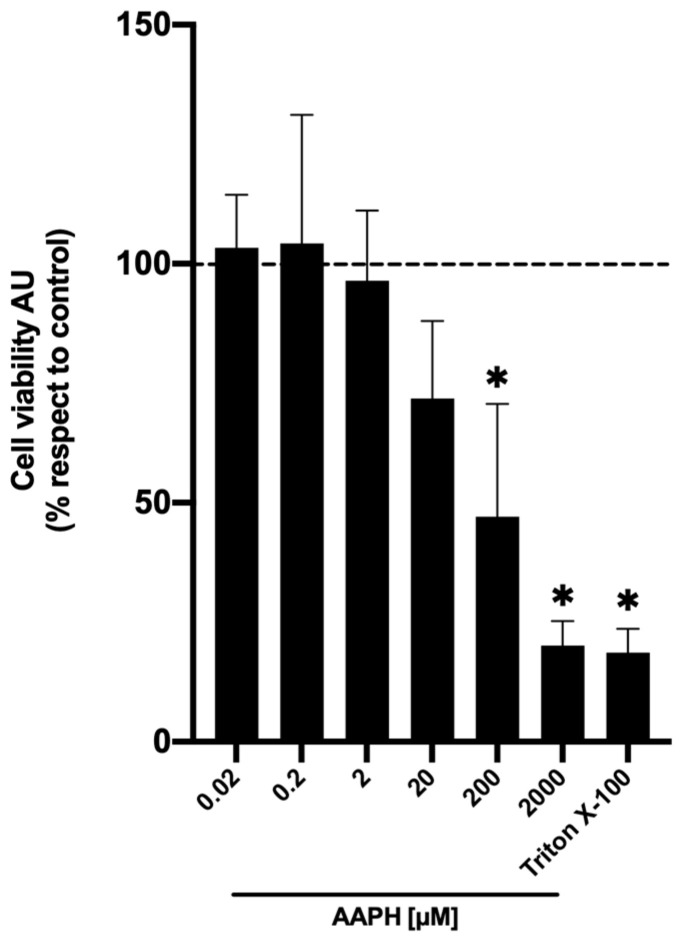
AAPH-induced cell death. Cell viability was evaluated by Alamar blue of HuH-7 cells treated with 2,2′-Azobis (2-amidinopropane) dihydrochloride (AAPH) for 24 h at different concentrations (0.02–2000 μM). For cell death control, cells were treated with Triton X-100 at 1% for 10 min. Data are expressed as a percentage of viability with respect to the control cells. Data are shown as mean ± SD (n = 3). AU, arbitrary units. A one-way ANOVA statistical test was performed, followed by the Tukey test. Statistically significant differences compared to the control group (cells without treatment) (* *p* < 0.05).

Potential hepatoprotective activity was determined in vitro under an oxidation damage assay induced by the oxidant AAPH. For in vitro hepatoprotection experiments, 200 µM of AAPH was used because this dose can cause close to 50% cell death (Figure 3) [38,39]. AAPH is an oxidant model frequently used for oxidation studies, since it could generate peroxyl radicals (ROO^•^) due to its spontaneous decomposition at 37 ± 1 °C [40,41]. Cell viability was evaluated using the Alamar blue assay.

**Figure 4:**
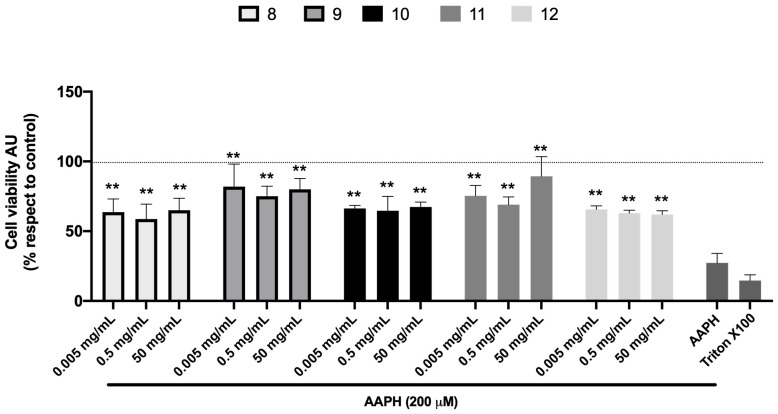
Honey extracts prevent AAPH-induced cell death. Cell viability was evaluated by Alamar blue of HuH-7 cells treated with quillay honey extracts (samples 8, 9, 10, 11, and 12) at different dilutions (0.005, 0.5, and 50 mg/mL at the final concentration in the medium) and co-treatment with 2,2′-Azobis (2-amidinopropane) dihydrochloride (AAPH) for 24 h at 200 μM. To control cell death, cells were treated with Triton X100 at 1% for 10 min. Data are expressed as a percentage of viability compared to the control group (untreated cells). AU, arbitrary units. Data are shown as mean ± SD (n = 3–5). A one-way ANOVA statistical test was performed, followed by the Tukey post hoc test. Statistically significant differences compared to the cells treated only with AAPH (** *p* < 0.01).

Hepatoprotective activity in vitro was determined under the same conditions as those used in the experiments to evaluate cytotoxicity. Samples 8 to 12 were selected due to their higher phenolic content and quillay percentage. HuH-7 cells were incubated for 24 h using three different dilutions (0.005, 0.5, and 50 mg/mL) of the honey extracts selected and co-treated with AAPH at 200 μM. Figure 4 illustrates the effects of honey extracts on AAPH-induced free radical accumulation at three different concentrations. The results confirmed that quillay tree honey extracts significantly protected against AAPH-induced damage. Cell viability increased substantially in the five honey-treated samples compared to cells exposed to AAPH alone (27.3% viability). The extracts showed a range of protective effects, with viability varying from 59% (0.5 mg/mL, sample 8) to 89% (50 mg/mL, sample 11). Samples 9 and 11 exhibited the most substantial protective effects, and were also among those with the highest antioxidant capacity according to the ORAC-FL assay.

## 3. Discussion

This study conducted a comprehensive characterization of Chilean honey samples through melissopalynological analysis, along with the evaluation of total phenolic and flavonoid content, antioxidant capacity, and hepatoprotective activity in HuH-7 cells. The profiles of phenolic acids and flavonoids from honey samples and quillay nectaries were also studied. Nectar composition is shaped by the environmental factors present in plants’ growing locations, and can vary across different locations [42,43,44,45]. Nectar production and composition variations can be influenced by various environmental factors, such as water availability, humidity, temperature, and light [42,43]. These environmental differences can alter the secondary metabolites present in the nectar, such as phenolic acids and flavonoids [42,43]. Biotic factors such as interactions with microbes and pollinators could also impact nectar composition [42,43]. Studies suggest that these variations are observed in regions with different climatic and microclimatic conditions [43]. For instance, the Mediterranean climate where quillay grows is characterized by well-defined wet and dry seasons, which affect the availability of water and environmental conditions, and can impact nectar composition.

Regarding the phenolic compounds, specifically phenolic acids (Table 2) and flavonoids (Table 3), we observed some differences between nectary and honey extracts. In the nectaries, chlorogenic acid, syringic acid, sinapic acid, ferulic acid, and phytohormone abscisic acid were identified, along with the flavonoids rutin, quercetin, pinocembrin, chrysin, and genistein. In honey, chlorogenic acid, syringic acid, *p*-coumaric acid, and abscisic acid were found in all samples, and the flavonoids galangin, pinocembrin, and chrysin were also consistently detected. Honey inherits secondary metabolites from the initial source plant species. Its properties are primarily attributed to these compounds, particularly phenolic compounds, which are responsible for a variety of biological activities in these products [46]. As these metabolites are transferred to honey by bees, the therapeutic properties of honey are directly linked to the plants that bees visit [20,21]. The phenolic acids and flavonoids identified in the honey samples (Table 2 and Table 3) are derived from quillay tree nectar and other plant species. This diverse botanical origin may explain the presence of compounds such as caffeic acid, *p*-coumaric acid, kaempferol, and galangin in the honey samples, despite their absence in quillay tree nectaries. Some phenolic compounds may also undergo changes in the process of nectar collection and its conversion into honey. The higher concentrations of certain compounds in nectaries, such as rutin, quercetin, or ferulic acid, compared to honey, could be due to their degradation by substances present within bee crops, such as *Lactobacillus* sp., leading to their lower concentration or absence in the final honey product [47,48,49].

Despite their botanical origin, plant species growing in Mediterranean climates are characterized by a high concentration of phenolic compounds, including phenolic acids and flavonoids. The environmental conditions in these regions promote the accumulation of these bioactive compounds, which are also reflected in the honey produced from these plants [50,51]. Studies on honeys from countries with Mediterranean climates, similar to the central zone of Chile where quillay grows, have shown elevated levels of total phenolic content (TPC) and total flavonoid content (TFC) [51]. For example, Italian honeys exhibited higher phenolic (210.5 mg GAE/kg honey) and flavonoid (50.8 mg QE/kg honey) content [48], while samples from Spain showed even higher values, with 1164.3 mg GAE/kg honey and 67.2 mg QE/kg honey for TPC and TFC values, respectively [52]. Aside from geographic and botanical origin, these variations may also result from different extraction methods or the nature of the samples used [53,54].

This is the first time that the Chilean unifloral quillay honey extracts have been studied in hepatic cell cultures using Human Hepatoma cells, HuH-7. The results demonstrated that quillay tree honey, rich in bioactive phenolic compounds, can protect and promote the survival of HuH-7 cells exposed to a generator of reactive oxygen species.

Concerning the cytotoxicity of honey, sample 9 was selected due to its high content of quillay pollen, which was representative of the unifloral quillay honeys analyzed. This allowed us to evaluate the biological activity of a sample with a strong presence of the target species, ensuring that any observed effects could be more closely associated with the phenolic and flavonoid compounds characteristic of quillay tree honey.

The bioactive compounds likely involved in the cytotoxicity and hepatoprotective activity are phenolic compounds, including phenolic acids and flavonoids [55,56]. Considering that the hepatoprotective activity of the honey was tested in cells with oxidative damage induced by free radicals, the antioxidant properties of these phenolic compounds are key contributors. This was demonstrated in the present study, where the honey samples containing these phenolic compounds exhibited significant antioxidant activity, suggesting that these compounds are responsible for the observed protective effects on hepatic cells.

The AAPH-induced oxidative stress model has been widely used to assess hepatoprotective potential via antioxidant mechanisms. In one study, AAPH-induced oxidative stress in Hepa1-6 cells demonstrated a significant increase in cell viability in the presence of bee pollen extracts compared to AAPH treatment alone, indicating that the extracts’ protective effects are linked to their ability to mitigate free radical accumulation and oxidative damage [38]. The model was also applied in HuH-7 cells treated with chickpea extracts, where all fractions exhibited hepatoprotective properties by reducing the oxidative damage induced by peroxyl radicals [39]. These studies underscore the relevance of AAPH as a hepatotoxic agent in oxidative stress models, where protection is closely related to the test compounds’ antioxidant potential.

In addition to the antioxidant properties, the honey extracts and its phenolic compounds examined here may also function through other mechanisms. Pre-treatment with chrysin significantly reduced the activity of liver function biomarkers and CCl_4_-induced TNF-α protein expression in mice (*p* < 0.001), suggesting that the hepatoprotective effect of chrysin may be mediated through the modulation of TNF-α processing, leading to reduced soluble TNF-α generation [57]. The flavone galangin has demonstrated protective effects against liver damage through multiple mechanisms. In one study, galangin inhibited CCl4-induced liver fibrosis in rats by scavenging oxygen free radicals, reducing lipid peroxidation, and inhibiting the activation and proliferation of hepatic stellate cells [58]. Another study showed that galangin also prevents cyclophosphamide-induced hepatotoxicity by activating Nrf2/HO-1 signaling, which attenuates oxidative damage, inflammation, and cell death, while increasing levels of glutathione and antioxidant enzymes [59]. Syringic acid has demonstrated hepatoprotective effects as well. In a rat model of thioacetamide-induced hepatic encephalopathy, it significantly reduced hepatotoxicity biomarkers while exhibiting strong anti-inflammatory and antioxidant properties [60]. Specifically, syringic acid mitigated oxidative stress by enhancing superoxide dismutase activity in liver tissues, replenishing glutathione levels, and reducing malondialdehyde and reactive oxygen species.

Several studies have outlined the bioactive roles of bee products in hepatoprotection. Chilean bee pollen extracts have been shown to reduce lipid accumulation in a steatosis model, highlighting their antioxidant and hepatoprotective properties [38]. Polish bee pollen extracts also showed a protective effect by reducing and/or preventing hepatic steatosis and degenerative changes acting on the liver of mice fed high-fat diets [61]. The effect of propolis on rats with NAFLD has been evaluated as well, revealing positive impacts on histopathological and biochemical parameters. These benefits were attributed to the antioxidant and anti-inflammatory properties of propolis [62].

The hepatoprotective effects of honey have been studied in various occasions. Honey has an inhibitory effect on liver cancer cells related to its antioxidant capacity. Honey treatment reduces nitric oxide levels and the number of cancer cells, enhancing its antioxidant profile. By reducing ROS and improving antioxidant activity, honey inhibited cancer cell proliferation. Honey also exhibited cytotoxic, antimetastatic, and anti-angiogenic effects on HepG2 cells [17,49]. The antioxidant and hepatoprotective effects of honey were evaluated in mice with paracetamol-induced liver damage as well, with results indicating hepatoprotection due to its antioxidant and/or perhaps pro-oxidative properties [56]. Buckwheat honey also significantly inhibited aspartate aminotransferase and alanine aminotransferase activities in mice with liver damage induced by carbon tetrachloride, showing a hepatoprotective effect primarily attributable to its high antioxidant capacity [63].

Researching the effects of honey on hepatic cells has gained significal attention, largely because of its antioxidant properties, which are primarily attributed to the presence of phenolic compounds [23,64,65,66]. These organic substances found in honey have been extensively documented for their remarkable antioxidant capacity. They play a pivotal role in neutralizing ROS [67,68], which are implicated in various hepatic disorders [14,16]. Our research is supported by evidence which suggests that these compounds may confer protective effects on the liver [14]. Our experimental results also contribute to the knowledge of a characteristic honey from Chile, providing empirical support for the positive influence of honey-derived phenolic compounds on hepatic cellular function.

## 4. Materials and Methods

### 4.1. Chemicals and Reagents

Unless otherwise specified, all reagents, HPLC-grade solvents, and standards were purchased from Sigma-Aldrich (St. Louis, MO, USA). Ferric chloride (FeCl_3_) and aluminum chloride (AlCl_3_) were purchased from Merck (Darmstadt, Germany). A MilliQ system was used for water purification (Synergy, Millipore, Darmstadt, Germany).

### 4.2. Samples

Twelve honey samples from six different regions of Chile were provided by associated beekeepers and stored at 2 °C until analysis. These samples came from the Valparaíso Region (33°02′47″ S 71°37′11″ W), Metropolitan Region (33°29′10.28″ S, 70°31′29.46″ W), Maule Region (35°25′36″ S 71°40′18″ W), Ñuble Region (36°37′00″ S 71°57′00″ W), BioBío Region (36°46′22″ S 73°03′47″ W), and Los Lagos Region (41°28′18″ S 72°56′12″ W). Botanical origin was determined via the melissopalynological analysis method as described in Chilean Standard NCH 2981.OF2005 [31] at the Botany Laboratory of the Plant Sciences Department in the Faculty of Agronomy and Forest Engineering, Pontificia Universidad Católica de Chile (Santiago, Chile). Quillay flower nectaries were collected in December 2023 in Santiago (33°29′10.28″ S, 70°31′29.46″ W), Metropolitan Region, Chile.

### 4.3. Honey and Nectary Extracts

Honey extracts were made as described by Ceslová et al. [69] with modifications. Five grams of each honey sample were diluted in 10 mL of ultrapure water, after which 15 mL of ethyl acetate, 15 mL of diethyl ether, and 150 μL of hydrochloric acid were added. After mixing in a magnetic stirrer for 20 min at room temperature, liquid–liquid extraction was performed. The mixture was transferred to a separatory funnel, and 15 mL of ethyl acetate was added. The funnel was shaken vigorously, and the top (organic) phase was collected in a clean container, while the extraction was performed from the aqueous (bottom) phase. This extraction process was repeated three times. Anhydrous sodium sulfate was then added to the organic phase to remove any residual water, and the solution was filtered using Whatman No. 1 paper. The solvent was evaporated under a vacuum at 38 °C. The final methanolic extract was obtained by dissolving the residue in methanol (2.5 g/mL) before being stored at −80 °C in the dark until use.

Nectaries were carefully removed from quillay flowers using a scalpel. Fourteen grams of nectary were mixed with 100 mL of methanol and crushed together in a mortar to ensure thorough extraction. The mixture was then stirred on a magnetic stirrer for one hour. Afterward, the solvent was collected in a clean container, and the procedure—crushing with 100 mL of methanol followed by stirring—was repeated. The solvents collected from both extractions were combined and vacuum-filtered. The resulting solution was then rotary evaporated until 8 mL remained, and stored at −80 °C in the dark until use, giving a final methanol extract (1.75 g/mL).

### 4.4. HPLC-DAD Analysis

Honey (2.5 g/mL) and nectary (1.75 g/mL) methanolic extracts were analyzed in a Hitachi Chromaster 5000 series HPLC instrument equipped with an autosampler and a photodiode array detector (Hitachi, Tokyo, Japan). The column used was a 250 × 4.6 mm Purospher STAR RP-18 end-capped with a similar guard column (Merck, Darmstadt, Germany). The Chromaster system manager V1.2 was used. An aliquot of 10 μL of the extracts was eluted in a mix of (A) methanol, (B) acetonitrile, and (C) 0.1% aqueous formic acid, with the following gradient elution: 0–10 min 20% B, 80% C; 10.1–40 min 7.5% A, 25% B, 67.5% C; 40.1–50 min 15% A, 25% B, 60% C, and 50–80 min 15% A, 50% B, 35% C. The flow rate was set at 0.8 mL/min, and the oven column was kept at 35 °C. A diode array detector (DAD) was used to measure the absorbance of the eluate (210–700 nm), and the chromatograms were integrated for both standards and extracts at 290 nm. Retention times of the standards and the UV-vis spectra were used for the identification. For quantification, a multistandard combination was also performed in equal concentrations of each compound (range 5–200 μM) to obtain calibration curves of all standards studied. All analyses were performed in triplicate for standards and samples. Data are expressed as mg/kg honey and mg/kg nectary [70].

### 4.5. Total Phenolic Content

The total phenolic content (TPC) was measured using the Folin–Ciocalteu method [71]. An amount of 125 μL of Folin–Ciocalteu reagent was mixed with 25 μL of honey extract, and 100 μL of Na_2_CO_3_ at 7.5% was added to each cell in 96-well polystyrene microplates. The samples were incubated for 60 min at 37 °C and measured at 765 nm in a microplate reader with stirring (AMR-100). Total phenolic quantification was performed using a gallic acid calibration curve (10 to 180 mg/L). Results were expressed as milligrams of gallic acid equivalents (GAE) per kg of honey (mg GAE/kg). Values are reported as means ± standard deviations (SDs) of three independent determinations.

### 4.6. Total Flavonoid Content

Flavonoid quantification was carried out according to Bridi et al. [70] with minor modifications. An amount of 105 μL of methanol was added to each of the 96-well polystyrene microplates, followed by 20 μL of honey extract and 125 μL of 2% AlCl_3_ solution. After 60 min at room temperature, the absorbance was measured at 420 nm in a 96-well plate Thermo Scientific Multiskan GO Spectrophotometer. Total flavonoid content (TFC) was calculated as milligrams of quercetin equivalents (QE) per kg of honey (mg QE/kg) from a calibration curve (20 to 180 mg/L), using methanol as a control. Values are reported as means ± SD of three independent determinations.

### 4.7. Ferric Reducing Antioxidant Potential (FRAP)

The ferric reducing power was determined as described by Bridi et al. [70] with some modifications. The working FRAP solution was mixed with 30 µL of the diluted extract (1.25 g/mL) and incubated in the dark at 37 °C for 30 min. Absorbance was measured at 594 nm in a microplate reader with stirring, AMR-100. An ethanol solution of Trolox (5–30 µM) was used as a positive control. Values were expressed as µmol Trolox equivalents per 100 g of honey (µmol TE/100 g), and are reported as means ± SD of 3 independent determinations.

### 4.8. Oxygen Radical Absorbance Capacity (ORAC)

The antioxidant capacity was determined according to Bridi et al. [72] with minor modifications for a fluorescent reader (BioTek™ Synergy™ Mx, Hampton, NH, USA, Laboratory Instrument). The fluorescein consumption was assessed by the decrease in fluorescence intensity of the sample (excitation 493 nm; emission 515 nm). AAPH (2,2′-azobis (2-amidinopropane) dihydrochloride) was used as the peroxyl ion generator, and μM Trolox as a standard (2–10 μM). Data are expressed as μmol Trolox equivalents per 100 g of honey (μmol TE/100 g). Values are reported as means ± SD of three independent determinations.

### 4.9. In Vitro Hepatoprotective Activity

Human hepatocellular carcinoma HuH-7 cells supplied by ATCC (American Type Culture Collection) were grown in 75 cm^2^ flasks using a Dulbecco Modified Eagle Medium (DMEM) with high glucose content, supplemented with 10% fetal bovine serum (FBS) and 1% antibiotic and antimycotic solution in a humidified atmosphere with 5% CO_2_–95% air at 37 °C. Sample 9 at different concentrations (0.005, 0.05, 0.5, 5, and 50 mg/mL) was used for the cytotoxicity assays. Cell culture occurred at 50,000 cells per well in 96-well plates, followed by a 24 h incubation. Viability was determined as the percentage of cell viability in relation to the control (untreated cell cultures). Cytotoxicity was determined by the reduction of resazurin (Alamar Blue Assay) and measuring fluorescence (560 nm excitation/590 nm emission) using a Cytation™ 5 multi-mode microplate reader from BioTek Instruments, Inc. (Winooski, VT, USA) in HuH-7 cells.

Five honey extracts at different concentrations (50, 0.5, and 0.005 mg/mL) were used for the hepatoprotective effect, and the cell viability was evaluated under the same conditions as explained above. Cell damage was induced using AAPH for 24 h (at 200 μM). Triton X-100 at 1% for 10 min was applied as a positive control of cell death. The results are expressed as a percentage of the control conditions of three independent experiments and six replicates per experiment.

### 4.10. Statistical Analysis

GraphPad Prism Software (9.0.1) and R Software (4.4.1) were used for all statistical analysis. Spearman correlations were performed with GraphPad Prism Software (9.0.1). Multiple group comparisons of continuous variables were performed with a one-way analysis of variance (ANOVA) followed by the Tukey post hoc test.

## 5. Conclusions

The present study helped to advance knowledge about unifloral quillay tree and multifloral Chilean honey. Our analysis revealed a correlation between the presence of quillay in honey samples and the phenolic compound content, and also showed the tendency toward a correlation between the samples with the highest percentage of quillay and the antioxidant capacity. The tests on HuH-7 cells confirmed the antioxidant potential and hepatoprotective effects of unifloral quillay honey, as shown by increased cell viability and antiradical activity against peroxyl radicals. Future studies exploring the detailed mechanisms underlying the hepatoprotective effects of quillay honey will be useful. In light of these results, our current studies also aim to evaluate the effects of quillay honey supplementation in the prevention and/or reversal of liver damage in a murine model of non-alcoholic fatty liver disease by assessing serum levels of liver damage markers (transaminases), dyslipidemia (triglycerides and cholesterol), and hepatic tissue steatosis, inflammation, and fibrosis.

## Figures and Tables

**Table 1:** Botanical origin and classification of honey samples.

Samples	Geographical Origin	Classification	Primary Species		Secondary Species		Tertiary Species	
			Species	%	Species	%	Species	%
1	Los Lagos Region	Unifloral	*Lotus pedunculatus* Cav. [Fabaceae]	73.8	*Weinmannia trichosperma* Cav. [Cunoniaceae]	8.5	*Caldcluvia paniculata* Cav. (D.Don) [Cunoniaceae]	7.1
2	Ñuble Region	Polyfloral	*Echium vulgare* L. [Boraginaceae]	18.5	*Quillaja saponaria* Molina [Quillajaceae]	17.0	*Amomyrtus luma* (Molina) D. Legrand & Kausel [Myrtaceae]	12.5
3	Metropolitan Region	Polyfloral	*Quillaja saponaria* Molina [Quillajaceae]	18.6	*Lotus pedunculatus* Cav. [Fabaceae]	13.8	*Lithraea caustica* (Molina) Hook et. Arn. [Anacardiaceae]	13.6
4	Maule Region	Polyfloral	*Quillaja saponaria* Molina [Quillajaceae]	23.5	*Echium vulgare* L. [Boraginaceae]	21.6	*Lithraea caustica* (Molina) Hook et. Arn. [Anacardiaceae]	8.4
5	BioBío Region	Bifloral	*Echium vulgare* L. [Boraginaceae]	25.9	*Quillaja saponaria* Molina [Quillajaceae]	23.9	*Lotus pedunculatus* Cav. [Fabaceae]	12.5
6	Maule Region	Polyfloral	*Quillaja saponaria* Molina [Quillajaceae]	30.8	*Escallonia pulverulenta* (Ruiz & Pav.) Pers. Escalloniaceae	14.3	*Lithraea caustica* (Molina) Hook et. Arn. [Anacardiaceae]	12.9
7	Valparaíso Region	Polyfloral	*Quillaja saponaria* Molina [Quillajaceae]	34.1	*Brassica rapa* L. [Brassicaceae]	16.3	*Lithraea caustica* (Molina) Hook et. Arn. [Anacardiaceae]	12.8
8	Metropolitan Region	Unifloral	*Quillaja saponaria* Molina [Quillajaceae]	50.2	*Lithraea caustica* (Molina) Hook et. Arn. [Anacardiaceae]	11.8	*Schinus latifolius* (Gillies Ex Lindl.) [Anacardiaceae]	6.9
9	BioBío Region	Unifloral	*Quillaja saponaria* Molina [Quillajaceae]	63.5	*Lotus pedunculatus* Cav. [Fabaceae]	9.1	*Lithraea caustica* (Molina) Hook et. Arn. [Anacardiaceae]	5.3
10	Maule Region	Unifloral	*Quillaja saponaria* Molina [Quillajaceae]	65.3	*Lithraea caustica* (Molina) Hook et. Arn. [Anacardiaceae]	10.8	*Schinus latifolius* (Gillies Ex Lindl.) [Anacardiaceae]	6.8
11	Valparaíso Region	Unifloral	*Quillaja saponaria* Molina [Quillajaceae]	65.4	*Lithraea caustica* (Molina) Hook et. Arn. [Anacardiaceae]	13.2	*Rubus ulmifolius* Schott [Rosaceae]	5.2
12	Maule Region	Unifloral	*Quillaja saponaria* Molina [Quillajaceae]	69.8	*Echium vulgare* L. [Boraginaceae]	6.6	*Brassica rapa* L. [Brassicaceae]	5.1

**Table 2:** Phenolic acids and abscisic acid of nectaries and twelve honey extracts quantified by HPLC-DAD.

Samples	Chlorogenic Acid	Cafeic Acid	Syringic Acid	*p*-Coumaric Acid	Sinapic Acid	Ferulic Acid	Abscisic Acid
Nectary	83.96 ± 2.24	n.d	52.67 ± 2.38	n.d	453.51 ± 8.32	1337.30 ± 25.54	33.85 ± 0.96
1	2.28 ± 0.01	2.79 ± 0.01	0.37 ± 0.01	0.16 ± 0.01	1.43 ± 0.16	n.d	11.75 ± 0.01
2	6.11 ± 0.05	n.d	0.44 ± 0.01	0.22 ± 0.01	2.82 ± 0.08	n.d	3.48 ± 0.04
3	7.23 ± 0.01	8.26 ± 0.01	0.39 ± 0.01	0.30 ± 0.05	1.11 ± 0.12	n.d	3.03 ± 0.02
4	4.38 ± 0.01	n.d	0.58 ± 0.01	0.21 ± 0.01	1.39 ± 0.05	n.d	18.71 ± 0.08
5	2.07 ± 0.03	n.d	1.06 ± 0.01	0.15 ± 0.01	n.d	10.16 ± 0.06	5.85 ± 0.02
6	3.26 ± 0.03	n.d	0.49 ± 0.01	0.19 ± 0.01	0.85 ± 0.05	0.36 ± 0.01	31.95 ± 0.01
7	2.48 ± 0.12	n.d	1.18 ± 0.01	0.26 ± 0.03	1.63 ± 0.05	n.d	9.02 ± 0.04
8	6.20 ± 0.02	n.d	1.43 ± 0.01	0.23 ± 0.05	1.72 ± 0.03	n.d	6.42 ± 0.06
9	6.26 ± 0.03	n.d	4.23 ± 0.03	0.10 ± 0.01	1.49 ± 0.05	n.d	8.13 ± 0.16
10	3.77 ± 0.14	n.d	0.72 ± 0.03	0.63 ± 0.01	2.24 ± 0.04	n.d	41.44 ± 0.25
11	8.58 ± 0.04	6.34 ± 0.15	0.60 ± 0.01	0.63 ± 0.03	n.d	11.36 ± 0.17	56.03 ± 0.26
12	3.92 ± 0.22	n.d	1.85 ± 0.01	0.39 ± 0.08	2.96 ± 0.04	n.d	12.49 ± 0.21

Data are expressed as mg/kg honey and mg/kg nectar, and the values represent the means ± SD (n = 3); n.d: not detected.

**Table 3:** Flavonoids of nectaries and honey extracts determined by HPLC-DAD.

Samples	Rutin	Quercetin	Kaempferol	Galangin	Pinocembrin	Chrysin	Genistein
Nectary	103.9 ± 4.89	451.34 ± 9.10	n.d	n.d	5.76 ± 0.08	1.04 ± 0.07	64.03 ± 1.18
1	10.77 ± 0.06	0.95 ± 0.02	1.09 ± 0.07	1.90 ± 0.05	1.07 ± 0.23	3.86 ± 0.15	0.76 ± 0.10
2	0.13 ± 0.01	0.67 ± 0.07	1.95 ± 0.21	2.39 ± 0.12	1.28 ± 0.13	5.05 ± 0.15	0.84 ± 0.11
3	5.99 ± 0.01	0.93 ± 0.01	16.35 ± 0.17	2.33 ± 0.03	1.27 ± 0.23	4.17 ± 0.36	n.d
4	2.39 ± 0.02	0.39 ± 0.01	1.55 ± 0.07	2.33 ± 0.06	1.31 ± 0.03	4.64 ± 0.06	0.97 ± 0.08
5	0.82 ± 0.03	0.16 ± 0.01	3.09 ± 0.03	2.83 ± 0.07	1.17 ± 0.04	4.77 ± 0.04	0.89 ± 0.06
6	6.71 ± 0.06	n.d	0.65 ± 0.03	1.97 ± 0.10	0.82 ± 0.13	3.54 ± 0.04	0.41 ± 0.02
7	7.67 ± 0.61	0.67 ± 0.15	5.43 ± 0.02	2.91 ± 0.06	1.27 ± 0.03	4.83 ± 0.08	1.01 ± 0.05
8	1.00 ± 0.01	n.d	1.89 ± 0.02	2.63 ± 0.05	1.18 ± 0.11	5.07 ± 0.35	1.12 ± 0.01
9	n.d	0.25 ± 0.01	1.63 ± 0.14	2.71 ± 0.14	1.43 ± 0.22	5.25 ± 0.22	0.93 ± 0.15
10	5.15 ± 0.03	0.21 ± 0.01	4.05 ± 0.09	2.14 ± 0.04	0.90 ± 0.05	3.97 ± 0.09	n.d
11	0.12 ± 0.01	n.d	n.d	2.08 ± 0.20	1.41 ± 0.01	6.00 ± 0.02	n.d
12	1.10 ± 0.04	n.d	2.78 ± 0.04	5.49 ± 0.27	2.24 ± 0.04	9.91 ± 0.15	n.d

Data are expressed as mg/kg honey and mg/kg nectary, and the values represent the means ± SD (n = 3); n.d: not detected.

**Table 4:** Quillay pollen percentage, TPC, TFC, and antioxidant activity of honey samples.

Samples	Quillay Pollen Percentage (%)	TPC (mg GAE/kg Honey)	TFC (mg QE/kg Honey)	ORAC-FL (μmol TE/100 g Honey)	FRAP (µmol TE/100 g Honey)
1	0	52.7 ± 8.5	7.4 ± 0.2	132.5 ± 7.9	3.6 ± 0.1
2	17.0	54.7 ± 10.6	11.1 ± 0.5	170.5 ± 1.4	4.4 ± 0.1
3	18.6	63.9 ± 9.9	20.7 ± 0.8	211.8 ± 14.5	5.0 ± 0.3
4	23.5	52.8 ± 9.6	9.9 ± 0.6	400.6 ± 7.4	6.8 ± 0.2
5	23.9	54.1 ± 7.5	9.8 ± 0.4	244.5 ± 4.0	4.1 ± 0.1
6	30.8	54.1 ± 9.7	9.6 ± 0.1	147.2 ± 7.8	4.1 ± 0.1
7	34.1	62.3 ± 12.3	13.8 ± 0.7	84.5 ± 8.0	4.2 ± 0.3
8	50.2	58.0 ± 11.3	11.8 ± 0.3	145.3 ± 10.4	3.6 ± 0.1
9	63.5	58.2 ± 11.6	8.9 ± 0.5	316.9 ± 10.0	4.1 ± 0.0
10	65.3	63.2 ± 10.3	11.9 ± 0.8	187.3 ± 6.3	3.9 ± 0.2
11	65.4	74.8 ± 12.6	12.2 ± 0.6	303.9 ± 5.9	8.1 ± 0.3
12	69.8	85.2 ± 14.1	16.8 ± 1.2	320.4 ± 6.3	5.8 ± 0.1

Data are expressed as mg/kg honey for TPC and TFC and mg/100 g honey for ORAC and FRAP. Values represent the means ± SD (n = 3).

## Data Availability

The data presented in this study are available on request from the first author.

## Supplementary Materials

The following supporting information can be downloaded at: https://www.mdpi.com/article/10.3390/plants13223187/s1, Figure S1: Spearman correlation analysis between the quillay pollen percentage and the chrysin concentration; Figure S2: Spearman correlation analysis between the quillay pollen percentage and the syringic acid concentration; Figure S3: Spearman correlation analysis between the quillay pollen percentage (%) and TPC (total phenolic content), TFC (total flavonoid content), ORAC-FL (oxygen radical absorbance capacity), and FRAP (ferric reducing antioxidant potential).
