# Supplementary material for: Comparative Study of Phenolic Content and Antioxidant and Hepatoprotective Activities of Unifloral Quillay Tree (Quillaja saponaria Molina) and Multifloral Honeys from Chile
Source: Plants (Basel). 2024 Nov 13;13(22):3187. doi: 10.3390/plants13223187 (PMC11597935; doi:10.3390/plants13223187)
Supplement: Supplementary file 1 [file plants-13-03187-s001.zip › plants-3263168-supplementary.pdf]

Comparative Study of Phenolic Content, Antioxidant, and Hepatoprotective Activities of Unifloral Quillay Tree (*Quillaja saponaria* Molina) and Multifloral Honeys from Chile

Paula Núñez-Pizarro , Gloria Montenegro , Gabriel Núñez , Marcelo E. Andia , Christian Espinosa-Bustos , Adriano Costa de Camargo , Juan Esteban Oyarzún, Raquel Bridi.

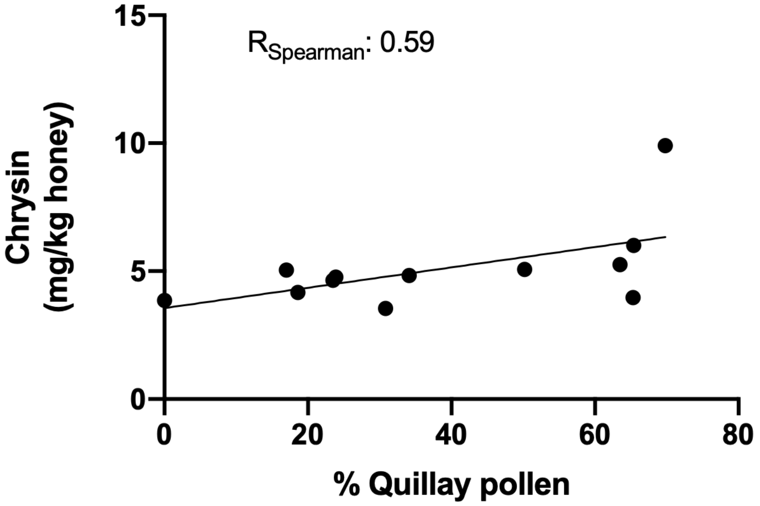

Figure S1. Spearman correlation analysis between the quillay pollen percentage and the chrysin concentration.

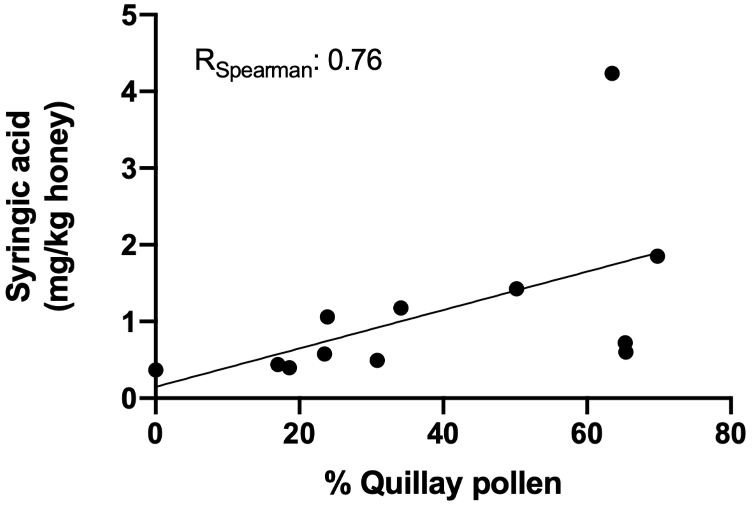

Figure S2. Spearman correlation analysis between the quillay pollen percentage and the syringic acid concentration.

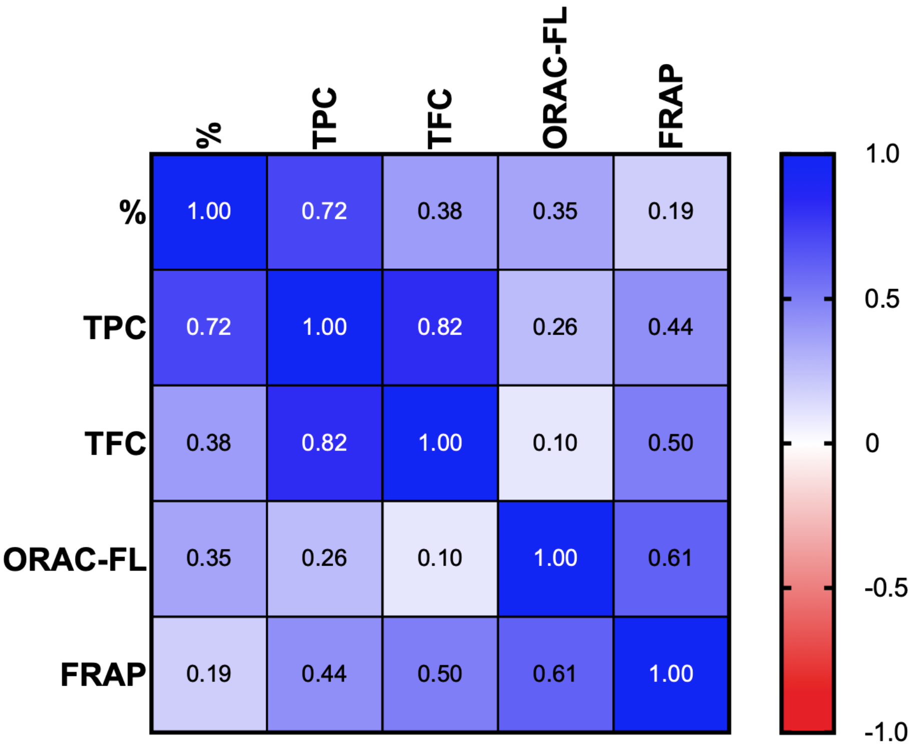

P values	%	TPC	TFC	ORAC-FL	FRAP
%		0.011	0.218	0.266	0.553
TPC	0.011		0.002	0.413	0.155
TFC	0.218	0.002		0.766	0.099
ORAC-FL	0.266	0.413	0.766		0.037
FRAP	0.553	0.155	0.099	0.037	

Figure S3. Spearman correlation analysis between the quillay pollen percentage (%) and TPC (total phenolic content), TFC (total flavonoid content), ORAC-FL (oxygen radical absorbance capacity), and FRAP (ferric reducing antioxidant potential).
